# Inactivation of the *WASF3* gene in prostate cancer cells leads to suppression of tumorigenicity and metastases

**DOI:** 10.1038/sj.bjc.6605850

**Published:** 2010-08-17

**Authors:** Y Teng, M Q Ren, R Cheney, S Sharma, J K Cowell

**Affiliations:** 1 MCG Cancer Center, School of Medicine, Medical College of Georgia, CN4112, 1120 15th Street, Augusta, GA 30912, USA; 2 Department of Pathology, Roswell Park Cancer Institute, Buffalo, NY 14263, USA; 3 Department of Pathology, School of Medicine, Medical College of Georgia, Augusta, GA 30912, USA

**Keywords:** WASF3, metastasis, prostate cancer, *in vivo* models, MMP

## Abstract

**Background:**

The WASF3 protein is involved in cell movement and invasion, and to investigate its role in prostate cancer progression we studied the phenotypic effects of knockdown in primary tumors and cell lines.

**Methods:**

ShRNA was used to knockdown WASF3 function in prostate cell lines. Cell motility (scratch wound assay), anchorage independent growth and *in vivo* tumorigenicity and metastasis were then compared between knockdown and wild-type cells.

**Results:**

Increased levels of expression were seen in high-grade human prostate cancer and in the PC3 and DU145 cell lines. Inactivation of WASF3 using shRNAs reduced cell motility and invasion in these cells and reduced anchorage independent growth *in vitro*. The loss of motility was accompanied by an associated increase in stress fiber formation and focal adhesions. When injected subcutaneously into severe combined immunodeficiency (SCID) mice, tumor formation was significantly reduced for PC3 and DU145 cells with WASF3 knockdown and *in vivo* metastasis assays using tail vain injection showed a significant reduction for PC3 and DU145 cells. The loss of the invasion phenotype was accompanied by down-regulation of matrix metalloproteinase 9.

**Conclusions:**

Overall, these observations demonstrate a critical role for WASF3 in the progression of prostate cancer and identify a potential target to control tumorigenicity and metastasis.

Metastasis is the primary cause of cancer death, and recent studies have shown that cancer progression to metastasis is controlled by specific genes, many of which are independent of the primary oncogenic transforming events. In this category of genes, some are related to metastasis suppression (Shevde and Welch, 2003; Steeg, 2003) and others are related to the promotion of metastasis (Sossey-Alaoui *et al*, 2007). We recently identified the *WASF3/WAVE3* gene at a chromosome translocation breakpoint involving the 13q12 region through a positional cloning strategy (Sossey-Alaoui *et al*, 2002) and subsequently showed that it is involved in the regulation of cell movement and invasion (Sossey-Alaoui *et al*, 2007) that was correlated with reduced lamellipodia formation at their leading edge (Sossey-Alaoui *et al*, 2005a, 2005b). As a result, *in vitro* invasion potential of these cells was drastically reduced.

The *WASF3* gene (Sossey-Alaoui *et al*, 2003) is a member of the Wiskott Aldrich syndrome family of proteins (WASP), which carry verprolin-cofilin-acidic domains at their C-terminal ends. These domains are thought to coordinate the recruitment of monomeric actin and the ARP2/3 complex of proteins to facilitate actin polymerization, which is essential for cell movement and invasion (Albiges-Rizo *et al*, 2009). Phosphorylation of WASP proteins has been shown to regulate their activity (Cory *et al*, 2002; Suetsugu *et al*, 2002; Torres and Rosen, 2003; Wu *et al*, 2004; Park *et al*, 2005) and enhances actin polymerization (Yokoyama *et al*, 2005). WASF2 was shown to be phosphorylated by the ABL kinase, which stimulated cell spreading and invasion (Stuart *et al*, 2006) and we recently showed that the same was true for WASF3 (Sossey-Alaoui and Li, 2007). Recently, WASF3 has been shown to localize to dendritic spines and constitutive activation of *RAC* resulted in translocation of WASF3 to the leading edges of lamellipodia guiding morphological plasticity of neurons (Pilpel and Segal, 2005).

During our overall survey of WASF3 expression in tumor cells, high levels were noted in prostate cancer cells and we now demonstrate that cells from high-grade tumors show increased WASF3 expression. We, therefore, initiated a series of experiments to analyze the role of WASF3 expression in prostate cancer metastasis. In this study we have demonstrated that transcriptionally stable knockdown of WASF3 in PC3 and DU145 cells using shRNA resulted in the inhibition of *in vitro* invasiveness and anchorage independent cell growth. Furthermore, knockdown of WASF3 significantly reduced xenograft tumor growth and metastasis *in vivo*. Thus, it appears that increased WASF3 expression could have a more general role in promoting metastasis in a variety of cancers that overexpress it, and so may be a valuable biomarker to predict tumor progression, as well as presenting a potential target, to prevent invasion and metastasis.

## Materials and methods

### Cell culture

Human PC3 and DU145 cell lines (American Type Tissue Collection) were cultured in RPMI-1640, supplemented with 10% fetal bovine serum (FBS, Hyclone, Logan, UT, USA), 10 U ml^−1^ penicillin, and 10 U ml^−1^ streptomycin, at 37°C in a humidified atmosphere containing 5% CO_2_.

### Retroviral vectors and transduction

The pSM2 retroviral vector containing a short hairpin RNA against WASF3 (Catalog no. RHS1764-9702604) was obtained from Open Biosystems (Huntsville, AL, USA). The Phoenix gag-pol only packaging cell line (ATCC) was transiently transfected with plasmid DNA using Effectene reagent (Qiagen, Valencia, CA, USA). The virus-containing supernatants were collected and filtered 48, 60, and 72 h after transfection and then used to infect PC3 and DU145 cell lines at least three times in the presence of polybrene (4 *μ*g ml^−1^, Sigma, St. Louis, MO, USA) and selected with 1.2 mg ml^−1^ puromycin (Sigma), respectively. Individual clones were obtained by ring-cloning and propagated in puromycin-containing media. To rescue WASF3 expression, full length of WASF3, lacking the 3′-untranslated region (UTR), was cloned into pCDNA3.1. WASF3-knockdown cells were transfected with either the empty pCDNA vector or pCDNA–WASF3 (pCDNA-W3). Following selection with G418, a mock population and WASF3-expressing stable cells were generated.

### Semi-Quantitative Reverse Transcription–PCR (RT–PCR) and Quantitative RT-PCR (qRT–PCR)

Total RNA was extracted using TRIzol (Invitrogen, Carlsbad, CA, USA) according to the manufacturer's instructions. To remove residual DNA, total RNA samples were treated with DNase I (Invitrogen). First-strand cDNA was synthesized using SuperScript II RT–PCR kit (Invitrogen) with 1 *μ*g of RNA. RT–PCR was performed using 1 *μ*l cDNA template to amplify the target genes with the following primers: WASF3 forward 5′-TGCCTTTAGTGAAGAGGAACA-3′ WASF3 reverse 5′-CAGCCCATCCTTCTTGTCAT-3′ WASF3 3′UTR forward 5′-CTTCAGCGTCTTTTCCTAGA-3′ WASF3 3′UTR reverse 5′-CAGGCTCATGAATATTTATG-3′ matrix metalloproteinase (MMP)-2 forward 5′-AGCTTTGACGATGACCGCAAATGG-3′, MMP-2 reverse 5′-GCCAATGGCTTGTCTGTTGGTTCT-3′ MMP-9 forward 5′-GAGGTTGACGTGAAGGCGCAGTG-3′, MMP-9 reverse 5′-ATAGCAGCTGCCTCAGTACT-3′ *β*-actin forward 5′-CCTCGCCTTTGCCGATCC-3′ *β*-actin reverse 5′-GGATCTTCATGAGGTAGTC-3′. RT–PCR was performed using a BioRad iCycler (Bio-Rad, Foster City, CA, USA) using BioRad iQ SYBR Green Supermix (Bio-Rad) with primers for WASF3 and GAPDH, respectively. Primers for human WASF3 were obtained from SABiosciences (Frederick, MD, USA) and primers for GAPDH were forward 5′-AATCCCATCACCATCTTCCA-3′ and reverse 5′-TGGACTCCACGACGTACTCA-3′. Experiments were performed independently at least three times and each sample was analyzed in triplicate. Gene expression levels were normalized against GAPDH.

### Immunoblot analysis

Cells were lysed in ice-cold RIPA buffer (25 mM Tris-HCl, pH 7.5, 150 mM NaCl, 1 mM EDTA, 1% Triton X-100, and a mixture of proteases inhibitors) and analyzed by SDS–PAGE followed by immunoblotting. Briefly, after blocking with 5% milk, the membranes were probed with primary antibodies at 4°C overnight. The membrane was washed and then incubated with secondary antibodies at room temperature for 1 h. Immunoreactive proteins were developed with enhanced chemiluminescence reagents (Pierce, Rockford, IL, USA). The following antibodies were used: rabbit anti-WASF3 (1 : 500, Cell Signaling, Danvers, MA, USA), mouse anti-*β*-actin (1 : 3000, Sigma). Horseradish peroxidase-conjugated anti-mouse and anti-rabbit were used as secondary antibodies (Pierce).

### Cell proliferation analysis

Cell proliferation activity was determined using the MTT method (4,5-dimethyl-thiazol-2yl)-2,5-difenyltetrazolium bromide). Cells were plated into 96-well plates at 3 × 10^3^ cells per well and cultured in 5% FBS growth media for various durations. At each end time point cells were treated with MTT (5 mg ml^−1^; Sigma) and incubated at 37°C and after 3 h the MTT solution was discarded and the wells were refilled with 200 *μ*l dimethyl sulfoxide. After mixing, the OD 560 value for each well was measured using a microplate reader (Bio-Rad).

### Wound-closure assays

DU145 cells were seeded into 6-well plates at 1 × 10^5^ per well. Confluent monolayers were starved overnight and a single scratch wound was created by dragging a 10 *μ*l plastic pipette tip across the cell surface. Cells were washed with phosphate-buffered saline (PBS) once to remove cell debris and supplemented with RPMI culture media containing 2% FBS. The area of a defined region within the scratch was measured using ImageJ software at time=0 and again after 24 h. The extent to which the wound had closed over 24 h was calculated and expressed as a percentage of the difference between times 0 and 24 h.

### Soft agar assays

Cells were seeded into 6-well plates using a two-layer soft agar system as described previously (Kunapuli *et al*, 2003; Sun *et al*, 2008). After 14 days incubation, colonies were stained with MTT for 30 min, and then the size and number of colonies was determined. All the experiments were repeated independently at least three times using triplicate plates.

### Matrigel invasion assay

Matrigel invasion assays were performed as described previously (Kunapuli *et al*, 2003). Briefly, transwells (BD biosciences, San Jose, CA, USA) with 8-*μ*m pore size filters covered with matrigel were inserted into 24-well plates. RPMI (500 *μ*l) containing 10% FBS was added to the lower chamber, and 200 *μ*l of a serum-free cell suspension (5 × 10^4^ cells) was placed in the upper chamber. The plates were incubated at 37°C with 5% CO_2_ for 24 or 36 h. After several rinses, the cells in the lower chamber were fixed in methanol and stained with 0.2% Crystal violet. Numbers of the invasive cells in nine randomly selected fields from triplicate chambers were counted in each experiment under a phase-contrast microscope.

### Animal experiments

Male 6- to 8-week-old mice were purchased from National Cancer Institute (NCI). Exponentially growing PC3 cells (2 × 10^6^ cells per flank) or DU145 cells (1 × 10^6^ cells per flank) were suspended in 100 *μ*l of serum-free RPMI per 100 *μ*l Matrigel (BD Biosciences) and injected subcutaneously into both flanks of SCID mice. First week after injection, the growth of primary tumors was monitored twice a week by measuring tumor diameters with calipers and calculating tumor volume (mm^3^) using the standard formula: *V*=(*L*+*W*^*2*^)/2, where *L* is the length and *W* is the width of a xenograft. Xenograft tumors were harvested at varying times after injection (see results) and individually weighed. Tumor samples were used for cell culture or for isolation of total RNA or protein. All animal experiments were approved by the MCG Animal Care and Use Committee.

To determine metastasis to the lung, 1 × 10^6^ PC3 or DU145 cells were injected into 6- to 8-week-old male SCID mice through the tail vein, respectively. Mice were killed 3 months after injection and the lungs were stained with 10% Indian ink through tracheal injection as described previously (Sossey-Alaoui *et al*, 2007). The lungs were then fixed in 10% neutral buffered formalin, embedded in paraffin blocks, sectioned at 5 *μ*m, and subjected hematoxylin and eosin staining.

### Immunofluorescence microscopy

DU145 cells were fixed with 3.7% formaldehyde in PBS for 15 min and permeabilized with 0.1% Triton X-100 in PBS for 10 min. After blocking for 1 h, the cells were incubated with anti-vinculin (Sigma) overnight at 4°C, followed by incubation for 1 h with fluorescein isothiocyanate goat anti-mouse IgG and Texas-red phalloidin (Molecular Probes, Eugene, OR, USA). Coverslips were mounted with Vectashield mounting medium (Vector Laboratories, Burlingame, CA, USA) containing nuclear stain 4′, 6-diamidino-2-phenylindole and then visualized using a Zeiss LSM 410 confocal microscope equipped with × 63 (1.4 numerical aperture) oil objectives (Carl Zeiss, Jena, Germany). Cells were scored positive for intensive stress fibers when bundles of actin filaments were seen clearly emerging from the central portion of the cell. In all quantifications, only those cells presenting with free borders were considered, and at least 100 cells from randomly selected fields were evaluated.

### Immunohistochemistry

Tumors and tissues were recovered by surgery and embedded in paraffin by routine diagnostic procedures. Paraffin sections were cut at 5 *μ*m, placed on charged slides and dried in a 60°C oven for 1 h. Room temperature slides were deparaffinized in three changes of Xylene and rehydrated using graded alcohols. Endogenous peroxidase was quenched with aqueous 3% H_2_O_2_ for 10 min and washed with PBS/T. Antigen retrieval was then performed using citrate buffer pH 6 in the microwave for 10 min, and allowed to cool for 15 min followed by a PBS/T wash. The slides were then placed on the DAKO (Carpinteria, CA, USA) autostainer and the following program was run: PBS/T wash followed by a 30 min incubation in 0.03% casein in PBS/T, and an 1 h incubation at room temperature with 0.5 *μ*g ml^−1^ of rabbit anti-WAVE3 (New England Peptide, Gardner, MA, USA). Rabbit IgG at 0.5 *μ*g ml^−1^ was used on a duplicate slide in place of the primary antibody as a negative control. A PBS/T wash was followed by biotinylated secondary goat anti-rabbit antibody for 30 min. A PBS/T wash was followed by the ABC reagent (Vector labs) for 30 min. Slides were washed in PBS/T and the chromogen 3, 3′-diaminobenzidine (DAKO) was applied for 5 min (color reaction product – brown). The slides were then counterstained with Hematoxylin, dehydrated, cleared, and coverslipped.

### Pro- and active MMP9 detection

PC3 or DU145 cells (1 × 10^5^) were seeded in 12-well plates and grown to 70–80% confluency in 1 ml of DMEM with 2% FBS. After 24 h, the media were collected in tubes and centrifuged for 10 min at 10 000 **g**. The pro- and active MMP-9 levels released into the media were measured using a Fluorokine MAP human MMP9 kit (R&D Systems, Minneapolis, MN, USA) according to the manufacturer's instructions. The fluorescent signal in the sample was determined using a BioRad analyzer at excitation/emission wavelengths of 340 nm per 465 nm.

### Statistical analysis

Where indicated, the results were representative of at least three independent experiments performed in triplicate and were expressed as the mean±s.d. Different values among groups were compared using the Student's *t*-test. For analysis of the *in vivo* metastasis data, statistical significance of the total number of tumors observed in DU145 experimental and control groups was assessed using a statistical test based on the Poisson model, which assumes that the number of tumors in a mouse is a Poisson random variate. This model is appropriate when small numbers of mice are present in both groups (Edgington, 1995). Under this model, and the null hypothesis of no difference across conditions, all Poisson variates in both groups have the same parameter. The difference between total number of tumors in control and experimental groups is; sum (control)-sum (test), which is a difference of two independent Poisson variates with parameters 2^*^mu and 4^*^mu following the so-called Skellam distribution (Karlis and Ntzoufras, 2006). As the value of mu may be computed from the data, the appropriate *P*-value can be obtained from Skellam distribution tables. For the analysis of PC3 experimental and control groups we used a standard permutation test (Edgington, 1995) to assess the significance of the observed number of tumors in the experimental group.

## Results

### High expression of *WASF3* in primary prostate cancer

**Figure 1:**
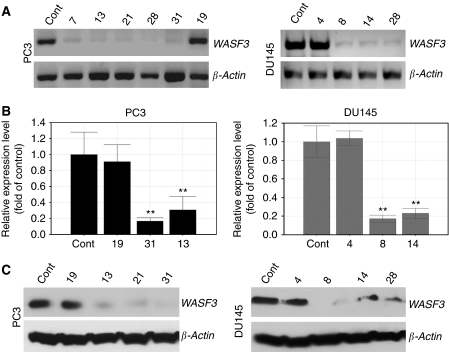
shRNA knockdown of WASF3 expression. (**A**) RT–PCR analysis of WASF3 mRNA expression shows high levels in parental (control) PC3 and DU145 cells. Expression levels are reduced in independent clones derived from PC3 (left panel) and DU145 (right panel) showing stable knockdown of WASF3. PC3 clone 19 and DU145 clone 4, however, retained parental levels of WASF3 expression. (**B**) Knockdown was confirmed in clones from both cell lines using qRT–PCR. ^**^P<0.01. (**C**) Western blot analysis of representative WASF3-silenced clones derived from either PC3 (left panel) or DU145 (right panel) shows highly reduced WASF3 protein levels. *β*-Actin was used as the loading control in this assay.

**Figure 2:**
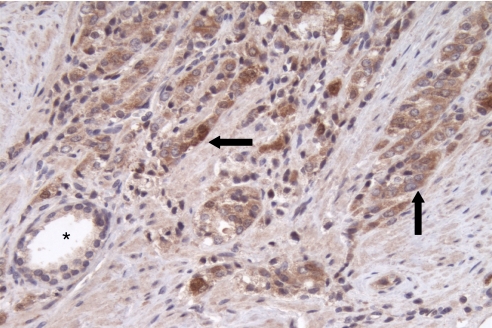
Immunohistochemical analysis of WAVE3 expression in prostate cancer. High levels of WASF3 in poorly differentiated prostatic adenocarcinoma (arrows) compared with minimal expression in normal prostatic ducts (^*^). (DAB immunohistochemistry; × 20 original magnification).

A survey of prostate cancer cell lines using qRT–PCR demonstrated that PC3 and DU145 cells (Figure 1) expressed readily detectable levels of WASF3, which correlated with protein levels as assessed using western blotting. Immunohistochemical analysis of high-grade human prostate cancer demonstrated increased levels of WASF3 compared with normal prostate epithelium (Figure 2). Thus, to investigate the consequences of inactivation of WASF3 in these prostate cancer cell lines, we created stable clones from PC3 and DU145 cells carrying shRNAs targeting WASF3.

### Knockdown of WASF3 in prostate cancer cells

PC3 and DU145 cells were transfected with shRNAs targeting the *WASF3* gene. Individual antibiotic-resistant clones were selected and analyzed for mRNA levels using RT–PCR and qRT–PCR. In the overall experiment, there was considerable variation in the level of the mRNA knockdown between clones, but several of them were shown to exhibit a high degree (>90%) of knockdown, which on western blotting showed a corresponding loss of protein (Figure 1). In addition, individual antibiotic-resistant clones from both cell lines were also identified that showed only marginal knockdown of expression and protein levels (Figure 1). These clones were used as additional controls in the biological assays described below.

Growth rates of these various clones were investigated using the MTT assay. No major difference in growth rates were seen in a comparison between PC3 or DU145 clones that showed high-level knockdown and clones that showed only marginal knockdown (data not shown).

### Loss of *WASF3* expression results in reduced *in vitro* invasion and motility

**Figure 3:**
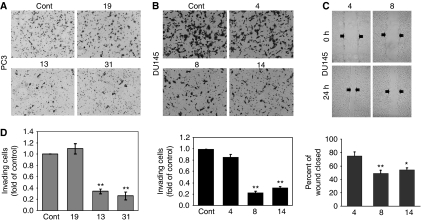
Knockdown of WASF3 expression impairs migration and invasion in prostate cancer cells. *In vitro* cell invasion was assessed using Boyden chamber assays. (**A**) PC3/shWASF3 clones (13 and 31) show significant (^**^P<0.01) reduction in invasion compared with PC3 parental (control), and clone 19, which shown normal levels of WASF3 expression. The same was true in a comparison of DU145 knockdown clones 8 and 14 (**B**). Wound-healing assays (**C**) show increased migration for DU145 clone 4 compared with knockdown clones 8 and 14 (**D**). Statistical values were ^*^P<0.05, ^**^P<0.01.

WASF3, and its family members, are involved in actin cytoskeleton reorganization and high level expression has been associated with invasion in breast cancer cells (Sossey-Alaoui *et al*, 2005a). To evaluate the consequences of loss of WASF3 protein expression in prostate cancer cells, we subjected them to matrigel invasion assays. The PC3 parental cells are highly invasive in this assay and freely pass through the membrane over a 24-h period. In contrast, the knockdown cells (Figure 3) clearly showed a significantly reduced invasion potential. The invasion potential for clone 19, which did not show knockdown of WASF3, was similar to the parental cells. DU145 cells showing >90% knockdown similarly showed a significant reduction in invasion potential, compared with cells that did not (Figure 3).

The scratch wound assay has also been used to characterize cell motility, which is another phenotype related to invasion. Unfortunately, the PC3 cells used in these studies have a rounded morphology and these cells tend to pile up in the dish rather than spreading out. As such, it was not possible to critically assess motility using the wound closure assay for these cells. DU145 cells, on the other hand, attach to the surface of the culture dish and spread out, allowing easy visualization of motility. When DU145 clone 4, which shows limited knockdown of WASF3, was compared with clones 8 and 14, which show extensive knockdown, a clear reduction in wound-healing capability was noted in the knockdown cells (Figure 3), further supporting the relationship between WASF3 expression and cell motility in prostate cancer cells.

These *in vitro* data clearly demonstrate that WASF3 can influence phenotypes related to tumor motility and invasion, suggesting that it may influence prostate cancer cell metastasis.

### Loss of WASF3 is associated with reduced anchorage independent growth

**Figure 4:**
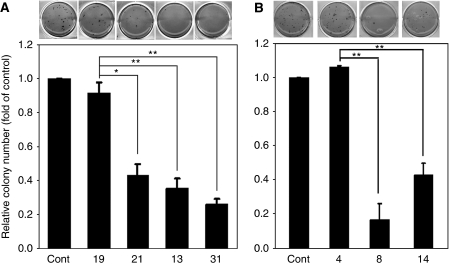
Knockdown of WASF3 expression reduces soft agar colony-forming ability in prostate cancer cells. Soft agar assays for PC3 (**A**) and DU145 (**B**) clones demonstrate reduced colony number (^*^P<0.05; ^**^P<0.01 respectively) for cells showing WASF3 knockdown (PC3 clones 21, 13 and 31 and DU145 clones 8 and 14) compared with cells that did not (parental cells (cont) and PC clone 19 and DU145 clone 4). Representative relative colony size for all of the experiments are shown in the petri dish views given above the histograms.

One of the hallmarks of malignant cells is their ability to grow under anchorage independent conditions. This phenotype is typically analyzed *in vitro* using soft agar clonogenic assays. To evaluate the role of WASF3 in anchorage independent growth, we seeded prostate cancer cells in soft agar and evaluated colony size and number (Figure 4). Parental PC3 cells form large colonies in soft agar with high efficiency, and WASF3-expressing PC3 clone 19 showed almost the same ability as the parental cells to grow in soft agar (Figure 4). In contrast, three knockdown clones, 13, 21, and 31, showed a significant reduction in colony number and size (Figure 4). In DU145 cells the same trend was observed. WASF3-expressing clone 4 was highly efficient in establishing clones in soft agar, whereas knockdown clones 8 and 14 were not. These data suggested that WASF3 might also have a significant role in tumor establishment.

### Loss of WASF3 is associated with reduced tumorigenicity *in vivo*

**Figure 5:**
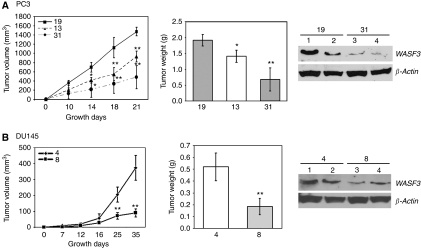
Knockdown of WASF3 expression suppressed the tumorigenicity of PC3 and DU145 cells in SCID mice. Growth curves for sub cutaneous tumor development *in vivo* show a significant reduction in tumor volume (mm^3^) and tumor weight (g) for the PC3 (**A**) and DU145 (**B**) knockdown clones over a 21 or 35-day observation period (^*^P<0.05 and ^**^P<0.01 by Student's *t*-test). Western blot analysis of tumors from four different mice for each clone (lanes 1 and 2 and lanes 3 and 4, respectively) from PC3 and DU145 (right) show persistent knockdown of WASF3 protein levels.

The *in vitro* analyses described above suggested that WASF3 can influence tumorigenicity and invasion of prostate cancer cells. To determine whether the same is true *in vivo*, 2 × 10^6^ cells from the shRNA knockdown clones 13 and 31 from PC3 were injected into the flanks of SCID mice (five animals per experimental group) in parallel with clone 19 that showed no knockdown of WASF3. Tumor development in control and knockdown cells was measured at regular intervals using calipers to assess tumor volume. In these studies, tumors from clone 19 developed rapidly reaching >1500 mm^2^ within three weeks (Figure 5A). In contrast, the two knockdown clones showed a significant reduction in tumor growth rate. In addition, analysis of the final tumor weights after 3 weeks demonstrated an overall significant reduction in the size of these tumors. Analysis of WASF3 protein levels in the resultant tumors demonstrated the maintenance of a significant reduction in WASF3 expression (Figure 5). Western blot analysis for WASF3 protein in representative tumors from clones 19 and 31 demonstrates that the protein levels in these tumors were similar to those seen in the implanted cells. When cells from these tumors were reintroduced into culture, and similarly analyzed, sustained knockdown of WASF3 was confirmed (data not shown).

When DU145 cells were analyzed for tumorigenicity in subcutaneous injections into both flanks of SCID mice, the same effects of WASF3 knockdown were seen. All of the mice injected with the control cells formed progressively growing tumors at both sites after 5 weeks after implantation (Figure 5B). In contrast, in the mice receiving the WASF3-knockdown cells, although 4 out of 5 mice developed tumors they were significantly smaller than those seen for WASF3-expressing cells (Figure 5). Western blot data demonstrated the maintenance of a significant reduction in WASF3 expression in these tumors (Figure 5). RT–PCR analysis of cells re-cultured from these DU145/shWASF3 tumor xenografts, further confirmed the persistent knockdown of WASF3 expression levels (data not shown).

### Down regulation of WASF3 protein reduces metastasis

**Figure 6:**
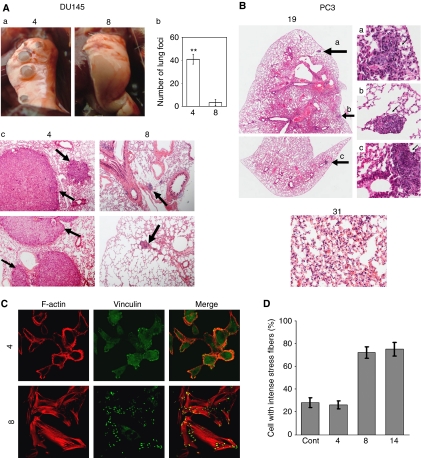
(**A**, **B**) Knockdown of WASF3 expression inhibits *in vivo* metastasis in SCID mice. Pulmonary metastasis of DU145 cells from clone 4 (no knockdown) show multiple surface tumors (**a**) unlike the knockdown clone 8 that shows vastly reduced numbers (**b**). Histochemical analysis of lungs from mice with clone 4 show large tumor foci in both cases (**c**). In lungs from the mice from clone 8 that developed surface tumors only small tumor foci were observed (**c**) (original magnifications: × 50). (**B**) Lungs from a mouse injected with cells from clone 19 (showing normal WASF3 levels) show multiple tumor foci in H & E-stained sections (right original magnifications: × 200), whereas lungs from mice injected with clone 31 did not show any tumors in hematoxylin and eosin-stained sections (original magnifications: × 50). (**C**, **D**) Knockdown of WASF3 expression increases the formation of stress fibers and focal adhesions in DU145 cells. The localization of F-actin and distribution of vinculin, a structural component of focal adhesions, was examined by indirect immunofluorescence using either an anti-vinculin or Texas-red phalloidin, antibody. In (**C**) clone 8 cells (original magnifications: × 630) show stress fibers and increased focal adhesions compared with non-knockdown clone 4. (**D**) The number of the cells with intensive stress fibers was increased ∼three-fold after knockdown of WASF3 expression. At least 100 cells were counted in each experiment and the values represent the mean±s.d. of triplicate experiments.

To investigate whether knockdown of WASF3 could affect metastasis of prostate cancer cells *in vivo*, as predicted from the invasion assays, we subjected selected clones to an experimental metastasis assay. DU145 cells from clone 4 were injected into the tail veins of three mice. One of these mice died and was lost to the study, but in the other two, 35 and 47 surface tumor nodules were counted (Figure 6). Histochemical analysis demonstrated tumor foci throughout the lungs in both mice (Figure 6). In contrast, of the four mice injected with cells from knockdown clone 8, only two showed lung surface tumors. In these mice there were fewer tumors (11 and 2, respectively), and they were smaller (Figure 6). The other two mice showed no tumor nodules. Using a parametric Poisson model to analyse these data demonstrates a highly significant difference between the two groups (*P*=0.0001). Histopathology analysis of the lungs from the mice injected with cells with normal expression levels of WASF3 revealed large tumors throughout the lungs (Figure 6). In lungs from the mice receiving the WASF3-knockdown cells, only infrequent, small tumor foci were seen in the mice showing surface nodules (Figure 6). In the other two mice no tumors were seen using histochemistry of sections through the lungs. PC3 cells from clone 31, which showed knockdown of WASF3, and clone 19 which did not, were similarly injected into the tail veins of SCID mice. After 12 weeks, the mice were sacrificed and the lungs removed for analysis. Two animals injected with clone 19 developed tumors that were clearly visible on the surface of the lung (data not shown). Histopathological analysis of these lungs also showed extensive infiltration of the apparently normal lung in these animals (data not shown). Three other animals that did not show visible tumors on the lung surface were also processed for immunohistochemistry analysis and lungs from two of them revealed microscopic tumor foci in the lungs (Figure 6). Thus, 80% of the control mice demonstrated metastases. In contrast, no tumors were identified in the lungs from the mice that received the clone with WASF3 knockdown on the surface or following histological analysis (Figure 6). Using a permutation test there was a significant difference in tumor incidence between the two groups (*P*=0.024).

### Loss of WASF3 expression increases stress fibers and focal adhesions

To understand the role of WASF3 in motility further, we investigated the reorganization of the cytoskeleton in DU145 cells carrying stable knockdown of the protein. Immuno-staining of DU145 parental cells show irregular membrane ruffles or abundant filopodia and lamellipodia that were typically accompanied by weak transverse actin stress fiber formation (Figure 6C and D). Using anti-vinculin antibodies, relatively few focal adhesion plaques were seen in these cells and were distributed apparently randomly. In cells showing stable knockdown of WASF3, however, extensive networks of stress fibers were seen and a more organized radial distribution of focal adhesion plaques were present along the leading edge of the cells (Figure 6C). Images from other knockdown clones are shown in Supplementary Figure S1. Quantitation of these observations showed that ∼80% of cells with WASF3 knockdown showed increased plaques and change in shape compared with ∼25% of cells showing normal WASF3 expression (Figure 6D).

### Loss of WASF3 results in down-regulation of MMPs

**Figure 7:**
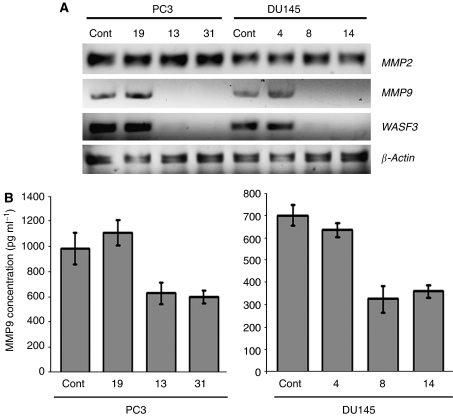
Knockdown of WASF3 expression suppresses MMP9 expression levels and inhibits MMP9 release from prostate cancer cell lines. (**A**) RT–PCR analysis of PC3 and DU145 cells showing knockdown of WASF3 also show down-regulation of MMP9, but not MMP2 expression levels. Cont=parental cells. (**B**) Secretion of the pro- and active form of MMP9 from PC3 and DU145 cells were measured in the supernatants of actively growing cells using the Fluorokine MAP assay procedure. Cells from each clone showing knockdown of WASF3 also showed reduced MMP9 levels compared with parental (control) and non-knockdown cells that did not.

As invasion and metastasis are associated with the expression levels of matrix metalloprotineases (MMP), we analyzed members of this protein family that have been implicated in prostate cancer progression. MMP9 showed reduced expression levels in clones from PC3 and DU145 with knockdown of WASF3, compared with control cells that did not (Figure 7A). Levels of MMP2 were unaffected by knockdown of WASF3 (Figure 7A). Analysis of the pro- and active forms of MMP9 released into the culture medium demonstrated reduced protein levels compared with the parental cells and clones with no knockdown of WASF3 (Figure 7B), consistent with a reduction in mRNA levels.

### Re-expression of a siRNA-resistant *WASF3* gene rescues the invasion and stress phenotypes

**Figure 8:**
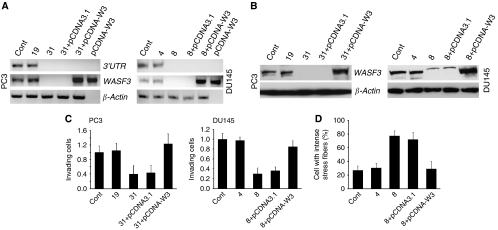
Re-expression of a siRNA-resistant *WASF3* gene rescues the invasion and stress response phenotypes in WASF3-knockdown cells. (**A**) PCR analysis of knockdown clones PC3 31 and DU145 8 using 3′UTR-specific primers do not recognize endogenous WASF3 transcripts in the clones transfected with the empty pcDNA3.1 vector or in the cells rescued with the WASF3 open reading frame (W3). Parental (cont) and WASF3-expressing clones #19 and #4, respectively, show robust expression of the endogenous WASF3 mRNA. The pCDNA3.1-W3 plasmid was used as a control for the PCR reactions. (**B**) Western blot analysis confirms the RT–PCR data. (**C**) Matrigel invasion assays demonstrate that the WASF3 rescued knockdown clones recover their invasion phenotype to a level that is not significantly different from the parental or control cells. (**D**) Stress fiber analysis of rescued cells demonstrates reduction in the number of cells showing stress fibers to levels similar to those seen in control cells.

To discount any potential off-target effects of the WASF3 siRNA use in these experiments we conducted rescue experiments of knockdown clones using a siRNA resistant WASF3 cDNA clone. The shRNAs used throughout this project target the 3′UTR of WASF3. We, therefore, constructed a full-length WASF3 cDNA in pcDNA3 that lacked the 3′UTR and would, therefore, be resistant to degradation in the WASF3-knockdown cells. This cDNA was introduced into clone 31 of PC3 cells and Clone 8 of DU145 cells. As shown in Figure 8A, using PCR primers that specifically amplify a region within the 3′UTR, neither the WASF3-knockdown clones nor the WASF3-rescued clones showed evidence of the full-length WASF3. Using primers that amplify a region within the WASF3 open reading frame, the rescued clones show WASF3 expression. These observations were confirmed by western blotting (Figure 8B). When these clones were challenged in the matrigel invasion assay (Figure 8C), the rescued clones showed almost the same ability as the parental cells to migrate through the matrix demonstrating recovery from the knockdown phenotype. When stress fiber formation was assessed in DU145 cells, the rescue cDNA resulted in a reduction in fibers to levels similar to those seen in the control cells (Figure 8D, see also Supplementary Figure S2).

## Discussion

Although prostate cancer is a common male malignancy, with current treatment options relatively few tumors progress to high-grade tumors in the lifetime of the individual (Lu-Yao *et al*, 2009). Thus, it is the tumors that are genetically programmed to progress that represent the major objective for drug targeting. Identification of genes, which facilitate progression, therefore, perhaps provides good targets for drug development. In this report we demonstrate the powerful influence of the *WASF3* gene on prostate cancer cell invasion and metastasis suggesting that its up-regulation in advanced stage human prostate cancer may offer a significant target. Because high-level knockdown (>90%, at the protein level) did not affect cell proliferation rates, we have described WASF3 as an invasion and metastasis promoter, although the *in vivo* studies clearly demonstrate that loss of WASF3 function also affects tumor development. It is possible that this effect is specific for xenografted tumors from cell suspensions, as these cells must rapidly reorganize into a tumor mass that likely requires significant involvement of actin cytoskeleton dynamics.

The demonstration that WASF3 promotes metastasis in prostate cancer cells, extends the previous observations for a single breast cancer cell line, MDA-MB-231 (Sossey-Alaoui *et al*, 2005a, 2007), although the lung metastasis assay is less robust for prostate cancer cells. We noted for parental PC3 cells, for example, that multiple lung nodules did not develop as seen for MDA-MB-231 cells, suggesting that this may not be an ideal assay for prostate cancer cell metastasis. Nonetheless, large surface tumors were seen in two (40%) of the experimental animals and multiple tumor foci were seen throughout the lungs destroying the cellular architecture. These animals, however, did not show any signs of respiratory distress despite the loss of lung tissue. In another two animals, the tumors were only observed following histopathological analysis and were relatively small. Significantly, none of the mice receiving the WASF3-knockdown clones showed any evidence of metastases on the lung surface or following histopathological analysis, reinforcing the role of WASF3 in promoting metastasis.

Degradation of the extracellular matrix by MMP activity is essential for many normal physiological processes, for example, during development, cell migration, growth and wound healing (Rodríguez *et al*, 2010). On the other hand, increased expression and activity of MMPs is also associated with tumor invasion, metastasis and angiogenesis (Basset *et al*, 1997; Westermarck and Kahari, 1999; Vincenti and Brinckerhoff, 2002). Expression of most MMPs is usually low in normal tissues, and induced when remodeling of the extracellular matrix is required. MMP expression is primarily regulated at the transcriptional level, although mRNA stabilization of MMP transcripts in response to growth factors and cytokines also has a role in the regulation of MMP activity (Johnsen *et al*, 1998). In prostate cancer cells, down-regulation of MMP9, but not MMP2 is consistent with the loss of the invasion/metastasis phenotype.
