# Supplementary material for: Inactivation of the WASF3 gene in prostate cancer cells leads to suppression of tumorigenicity and metastases
Source: Br J Cancer. 2010 Aug 17;103(7):1066–75. doi: 10.1038/sj.bjc.6605850 (PMC2965863; doi:10.1038/sj.bjc.6605850)
Supplement: Supplementary Information [file 6605850x1.doc]

**Supplemental Figure S1.**

**
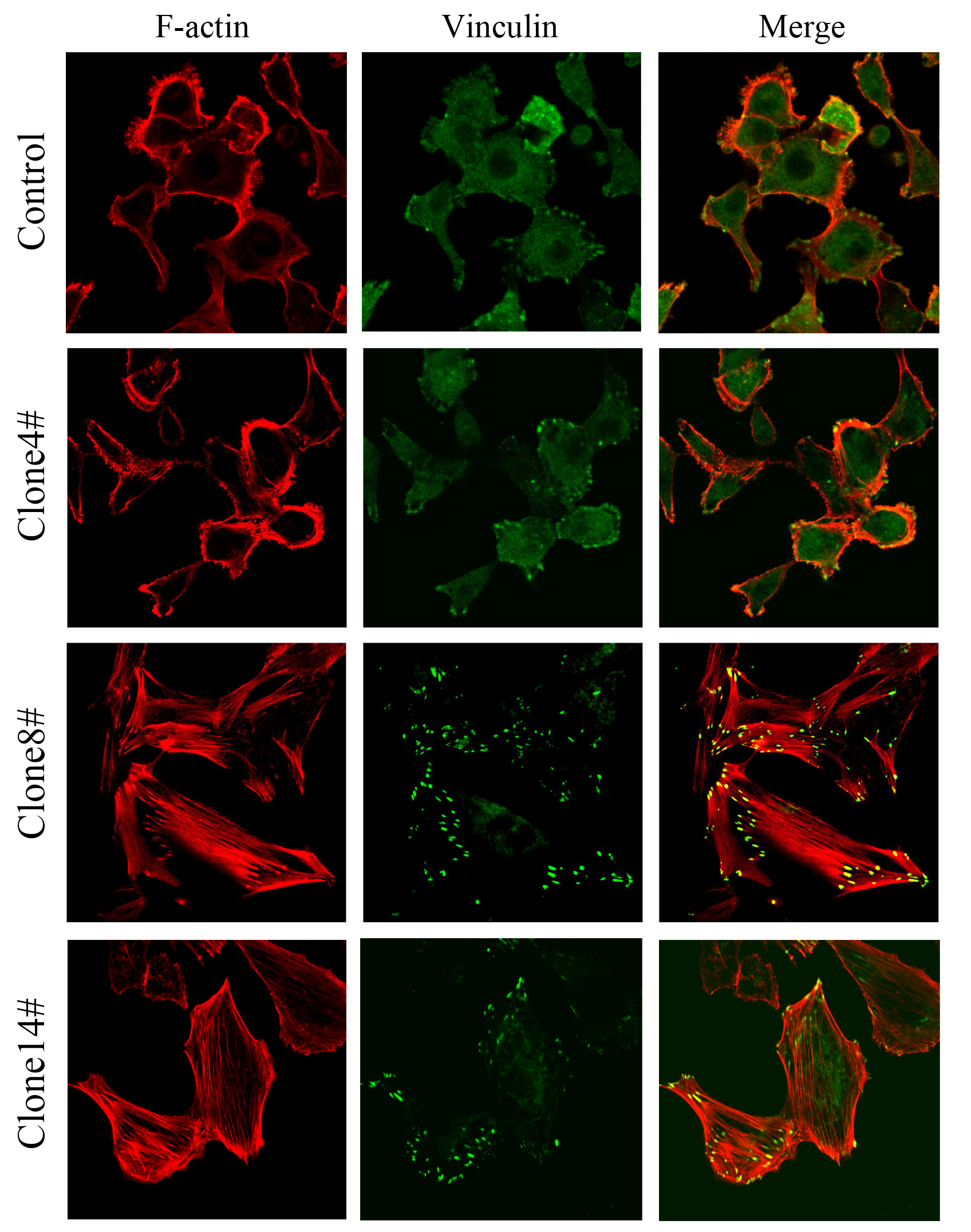
**

Examples of focal adhesion levels in knockdown clones #8 and #14 compared with non knockdown clone #4 and parental (control) DU145 cells.

**Supplemental figure S2.**

**
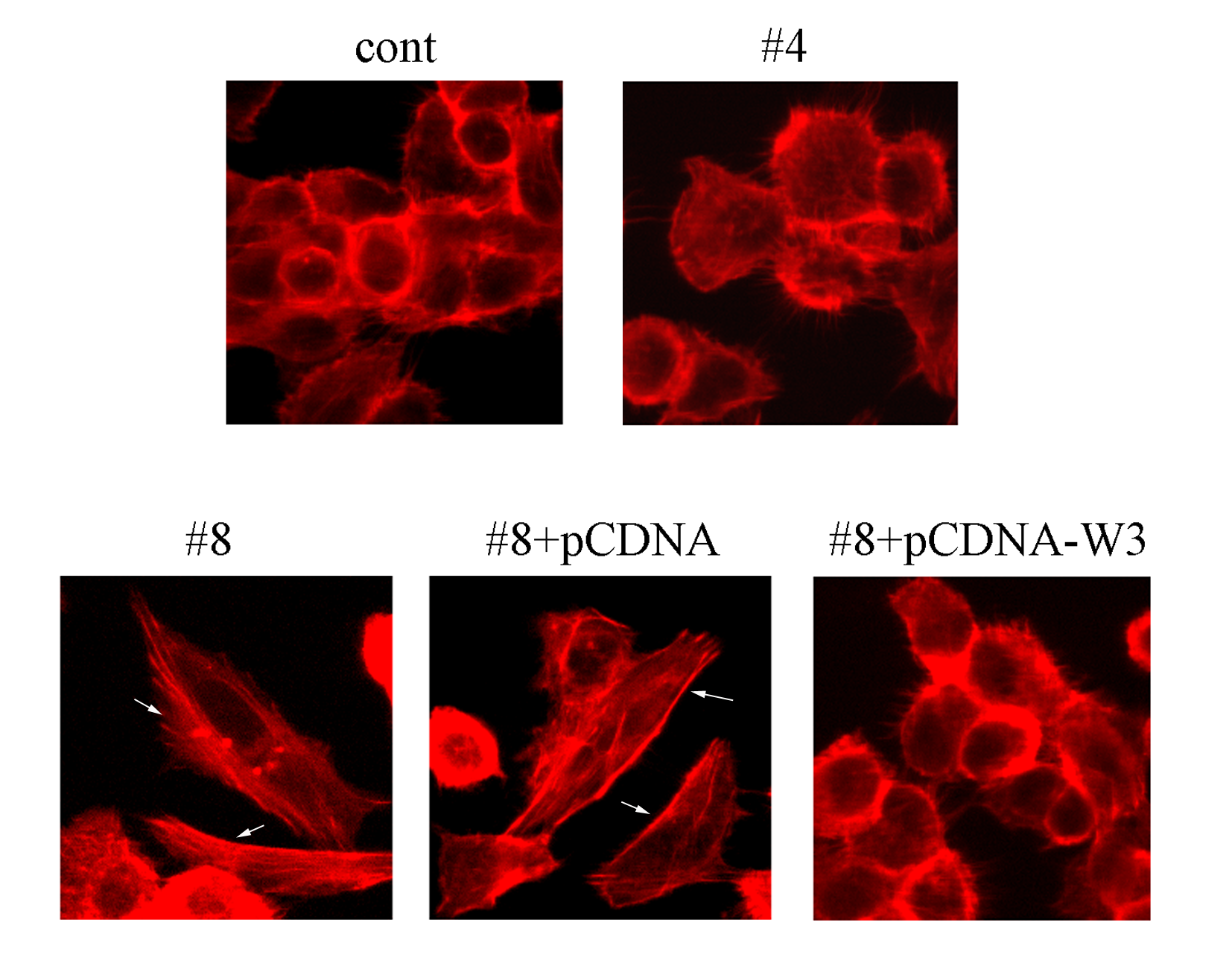
**

Parental DU145 cells (cont) show minimal presence of stress fibers and healthy lamellipodia formation at the surface of the cells. The same is true for clone #4 which did not show knockdown of WAVE3. In contrast WASF3 knockdown clone #8 shows increased frequency of stress fibers. Addition of the empty pCDNA3.1 to #8 cells does not reduce the frequency of stress fiber formation but when siRNA-resistant WASF3 is expressed exogenously in #8 cells, the frequency of stress fibers is reduced and the presence of lamellipodia at the surface is recovered.
